# Crossing Boundaries: Unraveling the Link Between Gender Dysphoria and Autism

**DOI:** 10.1192/j.eurpsy.2025.791

**Published:** 2025-08-26

**Authors:** C. M. Grego, A. I. Gomes, G. Simões, A. Tarelho

**Affiliations:** 1 Psychiatry and Mental Health Department, Local Health Unit of Aveiro Region, Aveiro, Portugal

## Abstract

**Introduction:**

Gender dysphoria (GD) and Autism Spectrum Disorder (ASD) are two complex conditions that can significantly impact an individual’s identity and well-being. Emerging research suggests a notable overlap between these two conditions, yet the nature and implications of this relationship remain underexplored. This paper aims to investigate the intersection of gender dysphoria and autism, considering both psychological and social dimensions.

**Objectives:**

The primary objectives of this study are to: (1) analyze the prevalence of gender dysphoria among individuals with autism, (2) explore the psychosocial factors contributing to the co-occurrence of GD and ASD, and (3) examine the implications for treatment and support strategies.

**Methods:**

A comprehensive literature search was conducted across multiple databases, including PubMed and Google Scholar, focusing on studies published between 2000 and 2024. Inclusion criteria centered on peer-reviewed articles that discuss the relationship between GD and ASD. Data were extracted and analyzed to identify common themes, prevalence rates, and psychosocial factors.

**Results:**

Preliminary findings indicate that individuals with ASD may experience higher rates of gender dysphoria compared to the general population. Factors such as social communication difficulties, rigid thinking patterns, and heightened sensitivity to social norms appear to influence the experience of gender identity. Qualitative data reveal that many individuals navigate significant challenges in accessing appropriate support and validation, often feeling marginalized within both the autistic and gender-diverse communities.

**Conclusions:**

This study highlights a critical intersection between gender dysphoria and autism, emphasizing the need for tailored support systems that acknowledge the unique experiences of individuals at this intersection. Increased awareness and understanding among healthcare providers can lead to more effective interventions and improved outcomes for those navigating both gender identity and autism. Future research should continue to explore this relationship to inform policy and practice, ultimately fostering a more inclusive environment for all individuals.

**Disclosure of Interest:**

None Declared

